# Problem-solving confidence, approach-avoidance style, and personal control among physical education undergraduates in Rwanda: a correlational study of differences by gender, instructional support, and year of study

**DOI:** 10.3389/fspor.2026.1865976

**Published:** 2026-09-28

**Authors:** Adalbert Dushimimana, Etienne Tugirumukiza, Etienne Twizeyimana, Aristide Rugamba, Pan YingXu

**Affiliations:** 1 Department of Volleyball Teaching and Research, School of Physical Education and Sports Coaching, Capital University of Physical Education and Sports, Beijing, China; 2 Department of Physical Education and Sports, College of Education, University of Rwanda, Kayonza, Rwanda; 3 Department of Track and Field Teaching and Research, School of Physical Education and Sports Coaching, Capital University of Physical Education and Sports, Beijing, China; 4 Department of Science Education, College of Education, University of Rwanda, Kayonza, Rwanda

**Keywords:** higher education, mixed-method study, physical education, problem-solving inventory, student’s perceptions

## Abstract

**Background/aim:**

Problem-solving confidence (PSC), approach–avoidance style (AAS), and personal control (PC) are recognized as key correlates of physical activity promotion and achievement in physical education. However, the extent to which these constructs differ by gender, perceived instructional support, and year of study among undergraduate students in resource-constrained African higher education settings remains insufficiently investigated. This mixed-methods study addresses this gap by examining these correlates in a sample of Rwandan physical education undergraduates.

**Methods:**

Data were collected through an online survey and subsequent semi-structured interviews. A total of sixty-five male and forty-five female students (*N* = 110) from a Rwandan university participated in the survey, with ten purposively selected for interviews. Factorial ANOVA and Tukey HSD *post hoc* tests were used to analyze differences in PSC, AAS, and PC by gender, perceived instructional support, and year of study. Thematic analysis of interview data provided additional qualitative insights.

**Results:**

Quantitative analyses indicated that PSC varied significantly by year of study (*p* = .035), as well as by the interactions of gender and year of study (*p* = .027) and gender, instructional support, and year of study (*p* = .025), but not by gender or instructional support independently. AAS differed solely by year of study (*p* = .044), with first-year students exhibiting the highest avoidance orientation. PC did not differ by gender, instructional support, or year of study individually, but was influenced by the interaction between gender and instructional support [F_(2, 90)_ = 3.443, *p* = .036, *η*^2^ = .071]. Qualitative findings revealed that inadequate infrastructure, insufficiently qualified instructors, and gender or cultural stigma impeded equitable participation and confidence development, particularly among female students.

**Conclusion:**

The three constructs demonstrate variation across specific combinations of demographic and instructional factors, rather than uniformly. Academic progression, particularly in interaction with gender and instructional support, is a decisive influence, with the first year identified as a critical period. These findings highlight the importance of implementing gender-sensitive, stage-appropriate instructional strategies and ensuring adequately resourced physical education environments. Enhancing personal control is essential for developing problem-solving skills, while addressing structural and cultural barriers to physical activity is necessary to prevent sedentary behavior and support both academic performance and personal well-being.

## 1 Introduction

### 1.1 Correlates unifying conception framework

This study investigates three interrelated dimensions of how undergraduate students engage with problems—problem-solving confidence (PSC), approach-avoidance style (AAS), and personal control (PC)—and examines how these dimensions vary with gender, perceived instructional support, and year of study among physical education undergraduates in Rwanda. Rather than treating these three dimensions as isolated psychological traits, the study is organized around a single, coherent conceptual model: the self-regulatory problem-solving model, which views PSC, AAS, and PC as mutually reinforcing components of one self-referential system of coping and agency.

In this model, personal control functions as the mediating core. The self-concept—an individual's organized perceptions of competence and worth across contexts (1)—gives rise to a generalized sense of personal control, defined as the intrinsic sense of worthiness and the perceived capacity to achieve personal goals (2, 3). This sense of control in turn shapes confidence: the belief that one can manage situational demands (4). Confidence then determines approach or avoidance: whether a challenging situation is appraised as exciting and manageable or as threatening and uncontrollable (5–7). Avoidance arises when a situation is perceived as uncontrollable, producing withdrawal and negative affect; approach emerges when confidence signals that the task can be mastered. The model therefore posits a directional logic that justifies the factorial analysis performed—that is from personal control (core) through problem-solving confidence (belief) to approach-avoidance style (behavioral engagement).

The Problem-Solving Inventory (PSI) developed by Heppner and Petersen (4) is uniquely well-suited to this framework because its three factors map directly onto the three components of the model: PSC captures confidence, AAS captures the behavioral engagement tendency, and PC captures the self-referential core of perceived control and self-worth. The three explanatory variables of this study—gender, instructional support, and year of study—are not treated as arbitrary correlates. Rather, each is hypothesized to enter the model at a specific point as a developmental and contextual input that shapes the core of personal control, which then propagates through confidence to approach-avoidance behavior.

### 1.2 From academic context to the problem-solving confidence

The relevance of this framework to higher education is immediate. A sedentary lifestyle is common among university students and is linked to depression, anxiety, and difficulty managing academic responsibilities (8, 9). Physical education (PE) students frequently counteract sedentary behavior through regular physical activity (10), an approach widely recognized as enhancing problem-solving confidence and fostering social problem-solving skills.

Across all fields, managing undergraduate workloads imposes significant demands that require advanced self-regulated learning and organizational skills. Effective problem-solving depends on higher-order cognitive processes that allow individuals to address complex, real-life situations (11). Operationally, this involves a calm acceptance and understanding of the issue, followed by the systematic application of relevant skills through four stages: defining and formulating the problem, generating alternative solutions, making decisions, and implementing and verifying the solution (12). A positive outlook is considered advantageous because it directly feeds the core of personal control, which recent research has made a central focus (13). As personal control increases, students' confidence is social problem-solving improves, and both the ability to address problems effectively and to maintain a positive approach develop progressively throughout educational levels (14). Promoting social problem-solving abilities has consequently been identified as a means of reducing the risk of mental health issues, including depression and diminished personal control.

### 1.3 Gender, personal control, and the Rwandan educational context

The study examines whether the core of personal control—and therefore the downstream components of confidence and approach orientation that it feeds—develops differently for male and female students. The existing literature reports group-level differences: some studies find that female students report lower problem-solving confidence than male students and display higher external motivation and demotivation when approaching problems (15, 16). It has also been shown that a stronger sense of personal control is associated with a reduced sense of inferiority, findings that hold for both men and women (17, 18).

Critically, however, this study does not interpret these observed differences as inherent or essential psychological characteristics of men and women. Rather, following the logic of the self-regulatory model, gender is treated as a socially and culturally situated variable that shapes access to the experiences—feedback, recognition, mastery opportunities, and freedom from stigma—through which personal control is built. Low problem-solving confidence has been linked to social stigma and to feeling undervalued in one's community (19–21). Where such barriers are distributed unevenly by gender, differences in PSC, AAS, and PC would be expected as a consequence of social position, not as a reflection of intrinsic psychological makeup.

This framing is especially pertinent to the Rwandan setting, where educational and cultural conditions shape how male and female students encounter academic demands, are recognized for competence, and negotiate self-presentation in learning environments. Rather than asserting that women generally possess poorer problem-solving confidence, the study examines whether gender differences emerge within specific educational and cultural context, and interprets any observed differences as situated outcomes amenable to change through institutional practice. The hypothesis is therefore stated cautiously: gender is expected to relate to PSC, AAS, and PC as far as the Rwandan educational context differentially shapes the experiences that build the personal control core.

### 1.4 Instructional support and the problem-solving confidence

The second explanatory variable, instructional support, is modeled as a contectual input that directly nourishes the core of personal control. Autonomy- and need-supportive teaching has been identified as a key determinant of student outcomes in undergraduate physical education (22, 23). Drawing on self-determination theory (SDT), Leo et al. (24). demonstrated that instructors who satisfy students' basic psychological needs for autonomy, competence, and relatedness promote higher self-determined motivation and improved adherence.

Within the self-regulatory framework, the roles of approach-avoidance theory and locus of control become interpretable as mechanisms rather than as competing constructs. SDT explains why autonomy support matters: It supplies the psychological nutrients that build perceived competence. Approach-avoidance theory (25) explains what follows: students with stronger perceived competence appraise challenges as opportunities and adopt approach-oriented engagement, while those with entrenched avoidance patterns—often fueled by a fear of negative judgment—require more individualized scaffolding before their engagement style shifts. Locus of control (26, 27) explains the individual difference in responsiveness: students with an internal locus of control thrive under autonomy-supportive conditions that afford agency, whereas those with an external locus of control persist more under guided, competence-building instruction that progressively transfers responsibility (4).

In this way the three theoretical frameworks are jointly integrated rather than merely juxtaposed: SDT specifies the interpersonal inputs, locus of control specifies the individual's responsiveness to those inputs, and approach-avoidance theory specifies the behavioral output. Together they predict that the effects of instructional support on PSI outcomes depend on students' existing levels of problem-solving confidence, approach-avoidance style, and perceived personal control—precisely the dimensions the PSI is designed to capture.

### 1.5 Year of study and the developmental trajectory of the problem-solving confidence

The third explanatory variable, year of study, captures the developmental dimension of the self-regulatory model. The level of undergraduate study—from the foundational first year to the specialized final years—significantly influences students' psychological resilience (28, 29), but the mechanisms underlying this association warrant scrutiny.

Early-stage undergraduates face the dual challenge of transitioning into higher education and managing novel academic demands, and tend to report higher levels of academic stress (30). Within the model, this acute stress acts on a still-fragile self-concept, frequently triggering an avoidance-oriented coping style—manifesting as academic procrastination, behavioral disengagement, or withdrawal as a defense mechanism. As students advance, however, accumulated academic exposure and repeated experiences of mastery cultivate stronger academic self-efficacy (31, 32). This construct mediates the transition from avoidance to approach-oriented coping: senior undergraduates come to confront complex, ill-structured problems with persistence rather than anxiety, to appraise challenges as opportunities for growth rather than as threats to competence, and to sustain engagement in the face of difficulty.

Consistent with the unifying model, resilience here is not treated as a fixed trait but as a developmental capacity evolving with academic maturity—the gradual strengthening of the personal control core across the undergraduate trajectory.

### 1.6 Study rationale and hypothesized model

This study builds on a single conceptual framework—a self-regulatory model in which personal control sits at the core, feeding problem-solving confidence, which in turn determines approach-avoidance engagement. Gender, instructional support, and year of study are not loosely related correlates but three distinct kinds of contextually and developmentally situated inputs into this system—social position, interpersonal environment, and developmental stage, respectively. The PSI's three-factor structure is not arbitrary but mirrors the three components of the model, and this is precisely why the study can test whether the three constructs—problem-solving confidence, approach-avoidance style, and personal control significantly differ by gender, instructional support, and year of study among physical education undergraduates in Rwanda.

The following three research questions were considered in this study

RQ1. How do problem-solving confidence, approach-avoidance style, and personal control significantly differ by gender among undergraduate physical education students?
RQ2. How do problem-solving confidence, approach-avoidance style, and personal control significantly differ by the level of instructional support among undergraduate physical education students?
RQ3. How do problem-solving confidence, approach-avoidance style, and personal control significantly differ by year of study among undergraduate physical education students?

## 2 Methods and design

A mixed-method study was conducted at a university in Rwanda focusing on physical education students across various education levels. The study used an explanatory research design to collect data from participants (33). A survey questionnaire was designed to capture perceptions of undergraduates in physical education and sports program regarding their problem-solving confidence, approach-avoidance style, and personal control. The aim was to investigate how problem-solving confidence, approach-avoidance style, and personal control differ by gender, levels of instructional support, and years of study. Quantitative data from the survey questionnaire were collected first and supplemented by qualitative data to provide in-depth insights that complement the numerical data from the quantitative investigation.

### 2.1 Sampling strategy

This study used a mixed-method approach with an explanatory research design, conducted among undergraduate students enrolled in a physical education and sports program. Participants were selected from four intact classes, representing all years from first to fourth in the undergraduate program. Purposive sampling included all students enrolled in the physical education module within these classes. Students participated voluntarily. Students were informed of their right to withdraw at any time without penalty, and no data from withdrawn participants were used unless provided before withdrawal.

### 2.2 Data collection instruments

Quantitative data were collected using the Problem-Solving Inventory (PSI) developed by Heppner and Petersen (4), which assessed participants’ perspectives on problem-solving confidence (11 items), approach-avoidance style (16 items), and personal control (5 items). Qualitative data were gathered through the researcher-designed interview guide. Participants' socio-demographic characteristics were also recorded. The PSI was adapted to the Rwandan higher education context. First, reliability was tested for three scales (PSC, AAS, and PC). Data analysis showed that all three scales had acceptable internal consistency. The problem-solving confidence scale (11 items) had a Cronbach's alpha of .903, indicating excellent reliability and that the items reliably measure the underlying construct. The approach–avoidance style scale (16 items) had a Cronbach's alpha of .890, reflecting good reliability with high inter-item consistency. The personal control scale (5 items) had a Cronbach's alpha of .846, also indicating good reliability, though slightly lower than the others. Overall, the instrument demonstrated strong internal consistency across all constructs, supporting its use for further analyses. Second, the interview guide consisted of three items assessing participation in reflecting on learning activities that require self-rating of PSI constructs, and two items requesting suggestions for future improvements. Participants' answers were coded using numbers and analyzed using themes and representative quotes. As depicted in Table 1, reliability was assessed using inter-coder agreement among three raters, yielding an overall consistency of 87.83% across the five items.

**Table 1:** Reliability test of the questionnaire used as the data collection instrument.

Variables	Items	Cronbach's alpha (*α*)	Interpretation
Problem-solving confidence	11	.903	Excellent reliability
Approach-avoidance style	16	.890	Good reliability
Personal control	5	.846	Good reliability

#### 2.2.1 Validity and reliability of instruments

Reliability and validity of the quantitative data were established through rigorous instrument testing and statistical verification. The Problem-Solving Inventory (PSI), developed by Heppner and Petersen (4) and adapted to the Rwandan higher education context, was assessed for internal consistency using Cronbach's alpha across all three subscales. The problem-solving confidence scale (11 items) demonstrated excellent reliability with an alpha of .903, while the approach–avoidance style scale (16 items) and personal control scale (5 items) yielded alpha values of .890 and .846, respectively (Table 1), confirming strong internal consistency and that the items reliably measured their underlying constructs. Content validity was supported by anchoring the instrument in a validated self-regulatory conceptual framework, and construct validity was reinforced by mapping the PSI's three-factor structure to the model. Data quality was further protected by reverse-coding the negatively worded items using a consistent transformation formula to ensure accurate scoring. Statistical validity was confirmed by testing key assumptions of factorial ANOVA, with Levene’s test of homogeneity of variances (PSC: *F* = 1.260, *p* = .244; AAS: *F* = 1.402, *p* = .164; PC: *F* = 1.738, *p* = .057) and the Shapiro–Wilk test confirming normality of the data (Table 2). The factorial ANOVA and Tukey HSD *post hoc* analyses were conducted on datasets meeting all necessary assumptions, strengthening the trustworthiness of the quantitative findings.

**Table 2:** Levene's test of equality of error variances^a^^,^b^^.

Variable	Basis of comparison	Levene statistic	df1	df2	Sig.
PSC variable	Based on mean	1.260	17	90	.673
AAS variable	Based on mean	1.402	17	90	.157
PC	Based on mean	1.738	17	90	.057

Tests the null hypothesis that the error variance of the dependent variable is equal across groups.

a Dependent variable: PSC, problem-solving confidence; AAS, approach avoidance style; PC, personal control.

b Design: Intercept + Gender + Instructional Support + Year of Study + ID × Instructional Support + Gender × Year of Study + Instructional Support × Year of Study + Gender × Instructional Support × Year of Study.

#### 2.2.2 Reliability and validity of qualitative data

Reliability and validity of the qualitative data were secured through the use of a researcher-designed interview guide and systematic, inter-rater-coded thematic analysis. The interview guide comprised items assessing participation in reflecting on learning activities that require self-rating of PSI constructs, together with open-ended items eliciting suggestions for future improvements, thereby ensuring the questions directly addressed the study's three constructs and supported content validity. Reliability was established through inter-coder agreement, with three coders analyzing participant responses across the five interview items and achieving an overall consistency of 87.853%, ensuring that the thematic interpretations were consistent and reproducible rather than idiosyncratic. Validity was enhanced by aligning the emergent themes directly with the study's constructs—problem-solving confidence, approach–avoidance style, and personal control—and by grounding findings in representative participant quotes that preserved authentic voices. The credibility of the analysis was further strengthened by methodological triangulation, in which qualitative themes such as inadequate infrastructure, unqualified instructors, and gender-based cultural stigma were systematically used to interpret and elaborate the quantitative results, demonstrating convergence that reinforces confidence in the overall interpretations.

#### 2.2.3 Data collection procedures

Data collection was carried out in two sequential phases reflecting the explanatory mixed-methods design. In the quantitative phase, an online survey was administered to undergraduate students enrolled in the physical education and sports program at a Rwandan university. The survey incorporated the adapted Problem-Solving Inventory (PSI) together with items capturing students' socio-demographic characteristics, including gender, level of instructional support, and year of study. A total of 110 students (65 males and 45 females) voluntarily completed the questionnaire, purposively drawn from four intact classes representing all 4 years of the undergraduate program. Before responding, students were informed of their right to withdraw at any time without penalty, and all participants provided consent. In the qualitative phase, semi-structured interviews were subsequently conducted with ten purposively selected participants to complement and elaborate the quantitative findings. The interviews followed a researcher-designed guide that explored students' experiences of inadequate infrastructure, unqualified instructors, and gender-cultural stigma in relation to the three constructs of problem-solving confidence, approach–avoidance style, and personal control. The interview sample was deliberately limited to ten participants to prioritize depth of information over statistical representation. All participants provided signed consent before taking part, and no data from withdrawn participants were used. This phased procedure ensured that qualitative insights could interpret and enrich the numerical results from the survey.

### 2.3 Data analysis

Quantitative data were primarily analyzed using Microsoft Excel for data organization and cleaning, and SPSS for statistical analysis after reversing negatively rated items. Descriptive statistics were calculated for three constructs: problem-solving confidence (11 items), of which constructs 3 and 10 were reversely coded; approach–avoidance style (16 items), of which constructs 1, 2, 3, 7, 8, 10, 13, and 15 were reversely coded; and personal control (5 items), all of which were reversely coded. Reverse coding was used because the constructs were negatively worded. To reverse a construct, a factor of 1 was added to the maximum score of the rating scale (9), followed by subtracting the original score from 10 as displayed by Equation 1.

NewScore=(MaximumValue+1)–OriginalScore (1)

Each construct was scored by averaging its item scores, with higher scores indicating higher levels of the construct. For inferential analysis, a factorial ANOVA was performed on the dependent variables against predefined correlates, such as gender, level of instructional support, and years of study, to assess their effects.

Data were checked for the assumptions of factorial ANOVA, including Levene's test for homogeneity of variances, for PSC, *F*_(15, 90)_ = 1.260, *p* = .244; for AAS, *F*_(15, 90)_ = 1.402, *p* = .164; and for PC, *F*_(15, 90)_ = 1.738, *p* = .057, satisfying the assumption of homogeneity of variances (Table 2) and the Shapiro–Wilk test for PSC, *W*_(110)_ = .981, *p* = .118, for AAS, *W*_(110)_ = .984, *p* = .195, and for PC, *W*_(110)_ = .991, *p* = .652, indicating the normality of the data (Table 3). After the data met these assumptions, analysis proceeded without further dataset transformation. Thematic analysis of interview data was used to deepen the understanding of participants' views on the three constructs. Coding followed an inductive approach, with three coders achieving an inter-coder agreement of 87.83%, and themes were aligned with the constructs.

**Table 3:** Tests of normality.

Variables	Kolmogorov–Smirnov^a^			Shapiro–Wilk		
	Statistic	df	Sig.	Statistic	df	Sig.
PSC variable	.073	110	.194	.981	110	.118
AAS variable	.074	110	.172	.984	110	.195
PC variable	.058	110	.200^b^	.991	110	.652

a Lilliefors significance correction.

b This is a lower bound of the true significance.

## 3 Results

This study examined gender, level of instructional support, and year of study as correlates of problem-solving confidence, approach-avoidance style, and personal control. The constructs of the problem-solving inventory items were measured on a rating scale ranging from 1 to 9 (Supplementary Appendix 7), including problem-solving confidence (*n* = 11), approach–avoidance style (*n* = 16), and personal control (*n* = 5).

To evaluate undergraduate physical education students' perspectives on problem-solving confidence, a factorial ANOVA, specifically a Three-way ANOVA, was conducted to compare main effects of gender, instructional support, and year of study as well as their interaction effects on problem-solving confidence. Table 4 presents the effects of a single correlate as well as the effects of the interaction among different correlates.

**Table 4:** Factorial ANOVA results for gender, instructional support, year of study, and their interactions as correlates of problem-solving confidence.

Tests of between-subjects effects						
Dependent variable: problem-solving confidence						
Source	Type III sum of squares	df	Mean square	*F*	Sig.	PES
Corrected model	41.983^a^	19	2.210	2.143	.009	.311
Intercept	1,985.159	1	1,985.159	1,925.335	.000	.955
Gender	11.455	1	11.455	11.110	.001	.110
Instructional support	8.068	2	4.034	3.913	.023	.080
Year of study	4.089	3	1.363	1.322	.272	.042
Gender × Instructional Support	5.106	2	2.553	2.476	.090	.052
Gender × Year of Study	2.621	3	.874	.847	.472	.027
Instructional Support × Year of Study	4.085	5	.817	.792	.558	.042
Gender × Instructional Support × Year of Study	9.152	3	3.051	2.959	.037	.090
Error	92.796	90	1.031			
Total	3,928.412	110				
Corrected total	134.779	109				

PSC, problem-solving confidence; PES, partial eta squared; MS, mean square; and Type III sum of squares.

a *R* squared = .311 (adjusted *R* squared = .166).

A three-way between-subjects factorial ANOVA (Type III sum of squares) was conducted to examine differences by gender, instructional support, and year of study on Problem-Solving Confidence. The overall model was significant, accounting for 31.1% of the variance in PSC. Significant differences emerged for gender (*p* < .05, *p* = .001); instructional support (*p* < .05, *p* = .023); and interaction among gender × instructional support × year of study (*p* < .05, *p* = .037), indicating that problem-solving confidence differs by gender and instructional support when considered as separate factors. No significant difference was observed for the year of study, interaction between gender and instructional support or between instructional support and the year of study.

To follow up on whether problem-solving confidence differs significantly by the correlates, namely instructional support and year of study, Tukey HSD *post hoc* comparisons were conducted to identify which correlate differed significantly. The problem-solving confidence emerged as independent of instructional support levels (Supplementary Appendix 1), whether minimal vs. moderate and high; moderate vs. minimal and high; or high vs. minimal and moderate (*p* > 0.05).

A one-way ANOVA revealed that PSC significantly differs by the year of study. *Post hoc* comparisons using the Tukey HSD test identified which year groups differed. The largest differences occurred between non-adjacent year groups. Year 1 students reported significantly lower confidence than Year 3 (*p* = .008) and Year 4 (*p* = .005). Similarly, Year 2 students scored significantly lower than Year 4 (*p* = .009). The comparison between Year 2 vs. Year 1 (*p* = .009) and Year 3 (*p* = .008) were statistically significant, and adjacent year pairs (Year 1 vs. Year 2, Year 3 vs. Year 4) showed no significant differences (Supplementary Appendix 2). These results suggest that meaningful gains in problem-solving confidence occur primarily across the broader span of the program rather than from 1 year to the next, with the most notable increases observed after the first year of study.

A three-way between-groups ANOVA was conducted to examine differences by gender, instructional support, and year of study on Approach-Avoidance Style (AAS). As shown in Table 5, differences by gender and instructional support were not statistically significant, and their differences were small to negligible. Similarly, most two-way and three-way interactions among these variables were not significant and had small differences. The only statistically significant main difference was the year of study (*p* < .05, *p* = .027). This indicates that AAS meaningfully differs across years of study, warranting further examination.

**Table 5:** Factorial ANOVA examining gender, instructional support, and year of study as correlates of approach-avoidance style.

Tests of between-subjects effects						
Dependent variable: approach-avoidance style						
Source	Type III sum of squares	df	Mean square	*F*	Sig.	PES
Corrected model	21.621^a^	19	1.138	1.322	.190	.218
Intercept	1,809.998	1	1,809.998	2,102.626	.000	.959
Gender	.949	1	.949	1.102	.297	.012
Instructional support	1.388	2	.694	.806	.450	.018
Year of study	5.798	3	1.933	3.945	.027	.070
Gender × Instructional Support	1.032	2	.516	.600	.551	.013
Gender × Year of Study	2.277	3	.759	.882	.454	.029
Instructional Support × Year of Study	3.738	5	.748	.868	.506	.046
Gender × Instructional Support × Year of Study	1.509	3	.503	.584	.627	.019
Error	77.474	90	.861			
Total	3,356.160	110				
Corrected total	99.096	109				

AAS, approach-avoidance style.

a *R* squared = .218 (adjusted *R* squared = .053).

Follow-up Tukey HSD *post hoc* comparisons (Supplementary Appendix 4) showed that the significant year difference was driven primarily by differences between first-year students and those in later years. Year 1 students reported the lowest AAS scores, and this difference was most pronounced when compared with Year 4 students (*p* < .05, *p* = .014). However, no significant differences emerged among students in Years 2, 3, and 4, suggesting that once students' progress beyond the first year, their Approach-Avoidance Style stabilizes and does not continue to differentiate across higher years of study. Detailed *post hoc* comparisons by instructional support are presented in Supplementary Appendix 3.

According to Table 6, the findings show that gender, instructional support, and year of study did not have significant main effects on personal control (PC)—indicating that none of these factors alone meaningfully predicts students’ sense of personal control. Only the interaction between gender and instructional support reached statistical significance, with a medium effect size [*F*_(2, 90)_ = 3.443, *p* = .036, *η*^2^ = .071]. This implies that the effect of instructional support on PC varies by gender; the same level of support may not benefit male and female students equally, pointing to the need for gender-aware support strategies. The remaining two-way and three-way interactions were all non-significant and yielded small effect sizes, and year of study did not meaningfully moderate any of these relationships. Tukey HSD *post hoc* comparisons further confirmed no significant differences in PC across instructional support levels or years of study (Supplementary Appendix 5, 6), suggesting that the significant interaction may reflect a more complex pattern—such as a crossover—rather than simple mean-level group differences.

**Table 6:** Factorial ANOVA examining gender, instructional support, and year of study as correlates of personal control.

Tests of between-subjects effects						
Dependent variable: personal control						
Source	Type III SS	df	MS	*F*	Sig.	PES
Corrected model	37.550^a^	19	1.976	.949	.526	.167
Intercept	805.658	1	805.658	387.044	.000	.811
Gender	.315	1	.315	.151	.698	.002
Instructional support	9.845	2	4.922	2.365	.100	.050
Year of study	1.051	3	.350	.168	.918	.006
Gender and instructional support	14.334	2	7.167	3.443	.036	.071
Gender and year of study	10.682	3	3.561	1.711	.170	.054
Instructional support and year of study	12.014	5	2.403	1.154	.338	.060
Gender, instructional support, and year of study	12.440	3	4.147	1.992	.121	.062
Error	187.341	90	2.082			
Total	2,742.040	110				
Corrected total	224.891	109				

a *R* squared = .167 (adjusted *R* squared = −.009).

### 3.1 Results from the interview

The qualitative phase followed the quantitative phase and involved interviews with 10 randomly selected participants to obtain in-depth information and supplement the quantitative data. These interviews were structured to explore participants' experiences related to the three variables under investigation (Supplementary Appendix 8). Analysis of the responses revealed three primary themes, each supported by representative quotes: (1) inadequate infrastructure, (2) unqualified Physical Education (PE) instructors, and (3) gender and cultural stigma. The sample size for interview participants was limited to 10 to prioritize depth of information over statistical representation and to better understand the underlying reasons for participants' experiences. As all participants were in a similar environment, the range of responses was not expected to be broad. Thus, a sample of 10 participants was deemed sufficient to provide comprehensive perspectives for this study.

### 3.2 Theme 1: inadequate infrastructure

………… “The inadequacy of our learning environment did more than test my physical skills; it challenged my fundamental belief in the possibility of solving problems. With bent hoops and faded court lines, each drill became an exercise in managing limited resources. I learned to adapt, but I also absorbed a more unsettling lesson: sometimes, the system is not designed to support your success, regardless of your preparation. This tension between resourcefulness and resignation has influenced my approach to every subsequent challenge, both in physical education and in academic settings.”

…………. “I usually encounter challenges of inappropriate courts for physical activities, equipment that is not specialized for a certain physical activity, and those available are insufficient.”

…………. “Shifting physical activity to simpler ones that do not demand the use of specialized equipment often becomes an alternative in case of a lack of specialized or insufficient equipment.”

All ten interviewed participants highlighted the inadequacy of existing infrastructure. In particular, they described unsuitable courts for physical activities and noted a lack of specialized equipment or equipment that was insufficient for specific activities.

Seven of the ten participants (70%) reported that when faced with infrastructure limitations, they opted for simpler physical activities that did not require advanced equipment. In contrast, three participants (30%) did not seek or use alternative solutions. Despite these differences, all participants demonstrated a strong willingness to engage in physical activities, indicating that when they have access to proper infrastructure, they participate actively.

### 3.3 Theme 2: unqualified PE instructors

…………. “In one instance, a substitute instructor assigned a complex team-based obstacle course but did not demonstrate the stations or clearly explain the rules. The instructions were vague, such as “just figure it out as you go” and the instructor remained passive rather than providing facilitation.”

This affected me in several ways. ……………… “Without clear demonstration or scaffolding, I expended more cognitive effort understanding expectations than on actual performance. The ambiguous task structure led to confusion regarding both the objective and the acceptable strategies.”

Regarding problem-solving, …………… “I became hesitant to commit to a plan because I lacked clear reference points for correct or safe execution. The lack of instructional guidance placed the entire risk-assessment burden on me, reducing my willingness to pursue novel solutions.”

All participants reported a heavy academic workload and the assignment of unrelated PE courses to inexperienced instructors. They also emphasized the lack of ongoing professional development and workshops for their PE instructors as key factors leading to students' ineffective participation in physical activities within the PE program.

### 3.4 Theme 3: gender and cultural stigma

……… “I frequently observe and experience gendered treatment in physical education classes. Activities are typically divided according to traditional gender stereotypes. Males are consistently encouraged to participate in competitive, high-intensity sports such as basketball and football, whereas females are directed toward activities perceived as more graceful or less physically assertive, including dance and aerobics. When I attempted to challenge this pattern, I encountered either surprise or subtle discouragement from instructors.”

………… “Some of my instructors assign complex tasks to male students instead of distributing tasks equally regardless of gender, and I consider this practice a form of gender-based bias in physical education. This practice equally limits my ability to engage in tasks of varying levels of complexity, negatively affecting my practical skills.”

……………… “Further factors that I observed exacerbating gender-based differences include instructors holding cultural beliefs in contexts where females are regarded as inferior to males, which becomes a barrier to inclusive practices and equitable participation in physical activity within a physical education course.”

Participants believed that gender becomes a barrier when the learning environment is not supportive for both male and female students. Nine out of ten participants (90%) pointed out that male students regularly participate in physical activity, while their female counterparts do not show consistent participation. Cultural norms that reserve certain physical activities for males were also highlighted as a barrier to inclusive and equitable participation in physical activity.

## 4 Discussion

This study investigated differences in problem-solving confidence (PSC), approach–avoidance style (AAS), and personal control (PC) by gender, perceived instructional support, and year of study among undergraduate physical education students in Rwanda. The results indicate that combinations of factors, rather than single correlates, exert greater influence, with the stage of a student's academic trajectory being most consequential. These findings suggest that the three constructs are developmentally shaped and contextually embedded, rather than fixed individual attributes.

### 4.1 Problem-solving confidence

Year of study was the only variable with a significant main effect on PSC. First-year students reported the lowest confidence, with substantial gains observed as students progressed through the program. However, these gains stabilized after the first year, as no significant differences were found among years 2, 3, and 4. This pattern suggests that the transition into higher education is the critical period for developing problem-solving confidence (14, 34). This finding is consistent with developmental literature on academic self-efficacy, which posits that accumulated mastery experiences and increased familiarity with the learning environment progressively strengthen students' confidence in addressing ill-structured problems (31). The significant two-way (gender × year) and three-way (gender × instructional support × year) interactions further demonstrate that these factors interact to shape PSC in ways not captured by individual correlates.

The lack of a significant main effect for perceived instructional support is noteworthy. This may indicate that students' perceptions of support, when measured at a single point in time, are less influential than the cumulative instructional relationships experienced throughout their studies. Qualitative accounts describing unqualified or passively supervising instructors, who “placed the entire risk-assessment burden on me, reducing my willingness to pursue novel solutions,” provide a plausible explanation. When instructional guidance is weak or inconsistent, perceived support may not reliably translate into experiences that build confidence.

### 4.2 Approach–avoidance style

AAS varied significantly by year of study, but not by gender or perceived instructional support. First-year students demonstrated the lowest, or most avoidance-oriented, scores, with the largest difference observed between first- and fourth-year students. After the fourth year, AAS scores stabilized. This finding aligns with evidence that early undergraduates often adopt avoidance-oriented coping strategies, such as procrastination, behavioral disengagement, and withdrawal, during the transition into higher education (14, 18). As students gain academic experience and mastery, they tend to shift toward approach-oriented coping (31, 35), a developmental transition reflected in the year-of-study gradient observed in the data.

### 4.3 Personal control

Unlike PSC and AAS, none of the three correlates produced a significant main effect on PC. The only significant finding was an interaction between gender and perceived instructional support, with a medium effect size [*F*_(2, 90)_ = 3.443, *p* = .036, *η*^2^ = .071]. This suggests that equivalent levels of instructional support do not benefit male and female students equally, highlighting the importance of gender-aware support strategies. Tukey HSD *post hoc* comparisons revealed no significant differences in PC across support levels or years, indicating that the interaction may reflect a more complex pattern, such as a crossover effect, rather than straightforward mean-level group differences.

The lack of a year-of-study effect on PC is notable, as it contrasts with previous evidence linking academic progression to improved personal control (36). This discrepancy may be due to limited opportunities for Rwandan physical education students to engage in learning tasks that require real-life problem-solving. Qualitative findings reinforce this interpretation, as inadequate infrastructure—such as bent hoops, faded court lines, and insufficient equipment—compelled students to adapt to resource constraints, described as “an exercise in managing limited resources,” which may undermine students' perceptions of systemic support for their success.

### 4.4 Gender and cultural context

The significant gender effects on PSC and AAS—alongside the gender × support interaction on PC—echo findings that female university students show lower levels of finding practical solutions in their learning and require greater external motivation to develop creative problem-solving skills (15). Our findings diverge from studies reporting a positive relationship between gender and PC (37, 38), a discrepancy plausibly attributable to contextual factors—personal characteristics, geographic location, facility availability, and cultural norms. The qualitative data provide the interpretive key: nine of ten interviewees reported that male students participate regularly while female students do not, and cultural beliefs that reserve certain activities for males were identified as barriers to equitable participation. That PE instructors “holding cultural beliefs in contexts where females are regarded as inferior” persist suggests that gender-based barriers are institutional and cultural rather than reflections of student interest.

### 4.5 Triangulation of quantitative and qualitative findings

The mixed-methods design allows the qualitative themes—inadequate infrastructure, unqualified instructors, and gender/cultural stigma—to interpret the quantitative results. The stability of PSC after the first year is consistent with participants’ accounts that confidence is shaped by accumulated, supported practice; the non-significant contribution of perceived instructional support is explained by the quality (rather than quantity) of that support; and the significant gender-related interactions are elaborated by documented patterns of differential participation. This convergence strengthens confidence in the interpretations offered above. The sedentary-lifestyle framing from the introduction is also underscored: students who cannot participate fully—owing to infrastructure limits or gendered exclusion—are unlikely to reap the confidence and control benefits of physical activity (9).

The findings indicate that developing problem-solving confidence and approach orientation in physical education undergraduates depends less on a uniform level of instructional support and more on stage-appropriate, gender-aware facilitation, enacted within adequately resourced environments.

## 5 Limitations

Although this study offers insights into physical education undergraduates' perceptions of problem-solving confidence, approach–avoidance style, and personal control, as well as their relationships to gender, perceived instructional support, and year of study, several limitations remain.

First, the cross-sectional design collected data at a single point in time and did not implement a long-term instructional support intervention. As a result, the study does not directly measure instructional support levels but instead assesses participants' perceived support. This design also precludes causal inference. Associations between year of study and the three constructs may reflect cohort differences or developmental changes, which only longitudinal follow-up can clarify.

Second, purposive sampling was used to select participants from intact classes. Although this approach aligns with the study's objectives, it may limit the generalizability of the findings to all physical education and sports students, particularly those at other Rwandan institutions or in other national contexts.

Third, the data rely exclusively on self-report instruments, which are susceptible to social desirability bias and to participants' subjective interpretations of constructs such as “instructional support.” The small qualitative sample (*n* = 10) also suggests that, although the interviews were thematically rich and showed high inter-coder agreement (87.83%), certain perspectives may have been excluded, potentially limiting the depth and nuance of the findings.

Fourth, data collection was limited to a single university, and the study did not include measures of actual physical activity, academic performance, or well-being. This limitation restricts the ability to link the three constructs to objective student outcomes. Finally, several effects, although statistically significant, showed small to medium effect sizes, and the factorial model explained only a limited proportion of variance in the outcome measures. Therefore, the findings should be interpreted with caution and not overgeneralized. Addressing these limitations will require randomized controlled trials or longitudinal intervention designs with large, randomly selected samples across multiple institutions.

## 6 Conclusion, recommendations, and directions for future research

This mixed-methods study examined whether problem-solving confidence, approach–avoidance style, and personal control differ by gender, perceived instructional support, and year of study among undergraduate physical education students in Rwanda. The results indicate that these constructs vary significantly across students’ academic progression and across their intersections with gender and instructional support. Specifically, problem-solving confidence differs by year of study and through its interactions with gender and perceived instructional support. Approach–avoidance style also varies by year, with first-year students exhibiting the highest avoidance orientation. Personal control differs only through the interaction between gender and perceived instructional support. Qualitative findings further identified inadequate infrastructure, insufficiently qualified instructors, and gender or cultural stigma as primary barriers to equitable and confidence-building participation.

Taken together, these findings suggest that academic progression, rather than instructional support alone, best explains variation in these constructs. Their development is sensitive to academic stage and is contextualized by gender, rather than uniform across the student population. The first year of study is a critical period for targeted intervention, and the quality of instructional support is more influential than its quantity. The results underscore the importance of personal control in facilitating problem-solving among physical education undergraduates. Furthermore, structural and cultural barriers to physical activity that contribute to sedentary behavior may negatively affect both academic performance and personal well-being.

Based on these results, the following recommendations are proposed: (1) establish adequate infrastructure to support students' active engagement in physical education programs; (2) provide ongoing professional development for physical education instructors to ensure familiarity with emerging physical activities and to adopt gender-inclusive, confidence-building pedagogies; and (3) address and challenge gender and cultural expectations that restrict equitable participation.

Future research should use longitudinal or experimental intervention designs to examine how students' problem-solving confidence, approach orientation, and personal control change with exposure to structured physical activities and gender-inclusive instruction. Studies investigating cultural perceptions of women's participation in physical activity, particularly those that enable participation without gender segregation, would provide additional insights. Further research linking these psychological constructs to objective measures of academic performance and well-being is also warranted. Such investigations would extend the preliminary findings identified in this study and inform evidence-based instructional design in higher education physical education programs.

## Data Availability

The raw data supporting the conclusions of this article will be made available by the authors, without undue reservation.
