# Supplementary material for: Problem-solving confidence, approach-avoidance style, and personal control among physical education undergraduates in Rwanda: a correlational study of differences by gender, instructional support, and year of study
Source: Front Sports Act Living. 2026 Sep 28;8:1865976. doi: 10.3389/fspor.2026.1865976 (PMC13656911; doi:10.3389/fspor.2026.1865976)
Supplement: Supplementary file 1 [file Supplementaryfile1.pdf]

Appendices

Appendix 1. **One-Way ANOVA Post-Hoc Comparisons**

Multiple Comparisons

Dependent Variable: Problem-Solving Confidence vs. Levels of Instructional Support

Tukey HSD

(I) Level of instructional support	(J) Level of instructional support	Mean Difference (I-J)	Std. Error	Sig.	95% Confidence Interval	
					Lower Bound	Upper Bound
Minimal	Moderate	-.3914	.32110	.445	-1.1566	.3738
	High	-.6669*	.25746	.060	-1.2805	-.0534
Moderate	Minimal	.3914	.32110	.445	-.3738	1.1566
	High	-.2755	.25746	.535	-.8890	.3380
High	Minimal	.6669*	.25746	.060	.0534	1.2805
	Moderate	.2755	.25746	.535	-.3380	.8890

Based on observed means.

The error term is Mean Square(Error) = 1.031.

*. The mean difference is significant at the .05 level.

Multiple Comparisons

Dependent Variable: Problem-Solving Confidence vs. Levels of Instructional Support

Tukey HSD

(I) Level of instructional support	(J) Level of instructional support	Mean Difference (I-J)	Std. Error	Sig.	95% Confidence Interval	
					Lower Bound	Upper Bound
Minimal	Moderate	-.0636	.37901	.985	-.9669	.8396
	High	-.3370	.30389	.511	-1.0612	.3872
Moderate	Minimal	.0636	.37901	.985	-.8396	.9669
	High	-.2734	.30389	.642	-.9976	.4508
High	Minimal	.3370	.30389	.511	-.3872	1.0612
	Moderate	.2734	.30389	.642	-.4508	.9976

Based on observed means.

The error term is Mean Square (Error) = 1.437.

Appendix 2 One-Way ANOVA Post-Hoc Comparisons

Multiple Comparisons

Dependent Variable: Problem-Solving Confidence vs. Year of Study

Tukey HSD

(I) Year of Study	(J) Year of Study	Mean Difference (I-J)	Std. Error	Sig.	95% Confidence Interval

					Lower Bound	Upper Bound
Year 1	Year 2	-1.1380*	.34981	.009	-2.0538	-.2223
	Year 3	-1.2246*	.37151	.008	-2.1971	-.2521
	Year 4	-1.8961*	.54862	.005	-3.3322	-.4600
Year 2	Year 1	1.1380*	.34981	.009	.2223	2.0538
	Year 3	-.0866	.26240	.988	-.7734	.6003
	Year 4	-.7581	.48148	.398	-2.0184	.5023
Year 3	Year 1	1.2246*	.37151	.008	.2521	2.1971
	Year 2	.0866	.26240	.988	-.6003	.7734
	Year 4	-.6715	.49746	.534	-1.9737	.6307
Year 4	Year 1	1.8961*	.54862	.005	.4600	3.3322
	Year 2	.7581	.48148	.398	-.5023	2.0184
	Year 3	.6715	.49746	.534	-.6307	1.9737

Based on observed means.
The error term is Mean Square(Error) = 1.437.
*. The mean difference is significant at the .05 level.

Appendix 3: One-Way ANOVA Post-Hoc Comparisons

Multiple Comparisons
Dependent Variable: Approach-avoidance style vs. Instructional support
Tukey HSD

(I) Level of instructional support	(J) Level of instructional support	Mean Difference (I-J)	Std. Error	Sig.	95% Confidence Interval	
					Lower Bound	Upper Bound
Minimal	Moderate	-.1906	.29340	.793	-.8898	.5086
	High	-.3152	.23524	.377	-.8758	.2454
Moderate	Minimal	.1906	.29340	.793	-.5086	.8898
	High	-.1246	.23524	.857	-.6852	.4361
High	Minimal	.3152	.23524	.377	-.2454	.8758
	Moderate	.1246	.23524	.857	-.4361	.6852

Based on observed means.
The error term is Mean Square(Error) = .861.

Appendix 4: One-Way ANOVA Post-Hoc Comparisons

Multiple Comparisons
Dependent Variable: Approach-avoidance style vs. year of study
Tukey HSD

(I) Year of Study	(J) Year of Study	Mean Difference (I-J)	Std. Error	Sig.	95% Confidence Interval

					Lower Bound	Upper Bound
Year 1	Year 2	-.2833	.25321	.679	-.9461	.3796
	Year 3	-.5133	.24444	.161	-1.1532	.1266
	Year 4	-.8407*	.27122	.014	-1.5507	-.1307
Year 2	Year 1	.2833	.25321	.679	-.3796	.9461
	Year 3	-.2300	.23453	.761	-.8439	.3839
	Year 4	-.5574	.26232	.153	-1.2441	.1293
Year 3	Year 1	.5133	.24444	.161	-.1266	1.1532
	Year 2	.2300	.23453	.761	-.3839	.8439
	Year 4	-.3274	.25386	.572	-.9919	.3372
Year 4	Year 1	.8407*	.27122	.014	.1307	1.5507
	Year 2	.5574	.26232	.153	-.1293	1.2441
	Year 3	.3274	.25386	.572	-.3372	.9919

Based on observed means.
The error term is Mean Square(Error) = .861.
*. The mean difference is significant at the .05 level.

Appendix 5: One-Way ANOVA Post-Hoc Comparisons

Multiple Comparisons
Dependent Variable: Personal control/low personal control vs. Instructional support
Tukey HSD

(I) Level of instructional support	(J) Level of instructional support	Mean Difference (I-J)	Std. Error	Sig.	95% Confidence Interval Lower Bound	95% Confidence Interval Upper Bound
Minimal	Moderate	-.6300	.45624	.355	-1.7173	.4573
	High	-.1243	.36581	.938	-.9960	.7475
Moderate	Minimal	.6300	.45624	.355	-.4573	1.7173
	High	.5057	.36581	.354	-.3660	1.3775
High	Minimal	.1243	.36581	.938	-.7475	.9960
	Moderate	-.5057	.36581	.354	-1.3775	.3660

Based on observed means.
The error term is Mean Square(Error) = 2.082.

Appendix 6: One-Way ANOVA Post-Hoc Comparisons

Multiple Comparisons
Dependent Variable: Personal control/low personal control vs. year of study
Tukey HSD

(I) Year of Study	(J) Year of Study	Mean Difference (I-J)	Std. Error	Sig.	95% Confidence Interval

					Lower Bound	Upper Bound
Year 1	Year 2	-.1281	.42109	.990	-1.2304	.9741
	Year 3	-.1878	.44721	.975	-1.3585	.9828
	Year 4	-.1467	.66041	.996	-1.8754	1.5821
Year 2	Year 1	.1281	.42109	.990	-.9741	1.2304
	Year 3	-.0597	.31586	.998	-.8865	.7671
	Year 4	-.0185	.57958	1.000	-1.5357	1.4986
Year 3	Year 1	.1878	.44721	.975	-.9828	1.3585
	Year 2	.0597	.31586	.998	-.7671	.8865
	Year 4	.0412	.59882	1.000	-1.5264	1.6087
Year 4	Year 1	.1467	.66041	.996	-1.5821	1.8754
	Year 2	.0185	.57958	1.000	-1.4986	1.5357
	Year 3	-.0412	.59882	1.000	-1.6087	1.5264

Based on observed means.
The error term is Mean Square(Error) = 2.082.

Appendix 7: Problem Solving Inventory (PSI) Questionnaire

The Problem Solving Inventory (PSI): A participant is required to rate their level of problem-solving skills (1-9) in comparison with other participants (1 = very much less, 9 = very much more; Heppner, 1979).

	**Problem-solving confidence**	1	2	3	4	5	6	7	8	9
1	I am usually able to think up creative and effective alternatives to solve a problem.									
2	I have the ability to solve most problems even though initially no solution is immediately apparent.									
3	Many problems I face are too complex for me to solve.									
4	I make decisions and am happy with them later.									
5	When I make plans to solve a problem, I am almost certain that I can make them work.									
6	Given enough time and effort, I believe I can solve most problems that confront me.									
7	When faced with a novel situation, I have confidence that I can handle problems that may arise.									
8	I trust my ability to solve new and difficult problems.									
9	After making a decision, the outcome I expected usually matches the actual outcome.									
10	When confronted with a problem, I am									

	unsure whether I can handle it.									
11	When I become aware of a problem, one of the first things I do is try to find out exactly what it is.									
	**Approach avoidance style**									
12	When a solution to a problem was unsuccessful, I do not examine why it didn't work.									
13	When I am confronted with a complex problem, I do not bother to develop a strategy for collecting information to define exactly what the problem is.									
14	After I have solved a problem, I do not analyze what went right or what went wrong.									
15	After I have tried to solve a problem with a certain course of action, I take time and compare the actual outcome to what I thought should have happened.									
16	When I have a problem, I think up as many possible ways to handle it as I can until I can't come up with any more ideas.									
17	When confronted with a problem, I consistently examine my feelings to find out what is going on in a problem situation.									
18	When confronted with a problem, I tend to do the first thing that I can think of to solve it.									
19	When deciding on an idea or possible solution to a problem, I do not take time to consider the chances of each alternative being successful.									
20	When confronted with a problem, I stop and think about it before deciding on a next step.									
21	I generally go with the first good idea that comes to my mind.									
22	When making a decision, I weigh the consequences of each alternative and compare them.									
23	I try to predict the overall result of carrying out a particular course of action,									
24	When I try to think up possible solutions to a problem, I do not come up with very many alternatives.									
25	I have a systematic method for									

	comparing alternatives and making decisions.									
26	When confronted with a problem, I do not usually examine what external factors in my environment may be contributing to it.									
27	When I am confused by a problem, one of the first things I do is survey the situation and consider all the relevant pieces of information.									
	**Personal control/low Personal control**									
28	When my first efforts to solve a problem fail, I become uneasy about my ability to handle the situation.									
29	Sometimes I do not stop and take time to deal with my problems, but just kind of muddle ahead.									
30	Even though I work on a problem, sometimes I feel like I am groping or wandering, and am not getting down to the real issue.									
31	I make snap judgments and later regret them.									
32	Sometimes I get so charged up emotionally that I am unable to consider many ways of dealing with my problems.									

Appendix 8: Interview

This interview was designed to elicit participants' experiences that map onto three themes namely (1) inadequate infrastructure, (2) unqualified PE instructors, and (3) gender and cultural stigma, while connecting them to the constructs under investigation including problem-solving confidence, approach–avoidance style, and personal control.

Researcher: Can you describe a time when facilities or learning space (e.g., equipment, courts, safety, availability of materials) were inadequate—how did that affect your confidence in solving problems during PE or learning tasks?

Participant:…………………………………………………………………………
……………………………………………………………………………………
…………………………

Researcher: When the environment was not supportive or resources were limited, what kinds of strategies did you rely on to figure things out, and how confident did you feel using those strategies?

Participant:…………………………………………………………………………
……………………………………………………………………………………
…………………………

Researcher: If you faced inadequate infrastructure, did you feel more likely to try again, explore solutions, and participate (approach), or to withdraw, avoid tasks, or give up (avoidance)? What led you to that choice?

Participant:……………………………………………………………………………
……………………………………………………………………………………………
…………………………

Researcher: How did the condition of the facilities (e.g., unsafe, crowded, poorly maintained) influence how you viewed your own competence or worth—did it strengthen or weaken your personal control?

Participant:……………………………………………………………………………
……………………………………………………………………………………………
…………………………

Researcher: Think about an instance when the PE instructor didn't seem trained, experienced, or well-prepared—how did that affect your ability to understand tasks and solve problems?

Participant:……………………………………………………………………………
……………………………………………………………………………………………
…………………………

Researcher: What specific kind of support (or lack of it) from the instructor helped or reduced your confidence in handling challenges—can you give an example?

Participant:……………………………………………………………………………
……………………………………………………………………………………………
…………………………

Researcher: When instruction was unclear or ineffective, did you tend to seek feedback and persist, or did you avoid trying and reduce your effort? What emotions or expectations shaped your response?

Participant:……………………………………………………………………………
……………………………………………………………………………………………
…………………………

Researcher: Have you experienced different treatment based on gender in PE or related learning contexts? How did that influence your confidence, willingness to attempt problem-solving, and personal control?

Participant:……………………………………………………………………………
……………………………………………………………………………………………
…………………………

Researcher: In your community or culture, are there beliefs or expectations that affect how certain genders should participate in PE or sports? How did those beliefs change whether you approached challenges or avoided them?

Participant:……………………………………………………………………………
……………………………………………………………………………………………
…………………………

Researcher: Looking across infrastructure issues, instructor support, and gender/cultural expectations, what do you believe most strongly shaped your personal control and your approach to solving problems—what would you recommend to improve your experience?

Participant:………………………………………………………………………………
………………………………………………………………………………………………
…………………………
